# Body mass index variation in adults with Williams syndrome: associations with predicted dietary intake and food behaviors

**DOI:** 10.29219/fnr.v67.9321

**Published:** 2023-06-23

**Authors:** Danielle Renzi, Takara Stanley, Jessica Waxler, Hang Lee, Barbara Pober, Marianne Nordstrom

**Affiliations:** 1 Division of Genetics, Department of Pediatrics, Massachusetts General Hospital, Boston, MA, USA; 2 Metabolism Unit, Department of Medicine, and Pediatric Endocrine Unit, Department of Pediatrics, Massachusetts General Hospital, Boston, MA, USA; 3 Harvard Medical School, Boston, MA, USA; 4 Biostatistics Center, Massachusetts General Hospital, Boston, MA, USA; 5 Department of Nutrition, Institute of Basic Medical Sciences, University of Oslo, Oslo, Norway; 6 Frambu Resource Centre for Rare Disorders, Siggerud, Norway; 7 Unit for Rare Neuromuscular Disorders, Movement, Muscle and Neurodegeneration, Department of Neurology, Oslo University Hospital, Oslo, Norway

**Keywords:** Williams syndrome, body weight, food frequency questionnaire, food behavior, weight trajectory

## Abstract

**Background:**

Dietary intake and body weight are important predictors of long-term health. However, few studies have focused on these topics in adults with genetic syndromes that have associated intellectual disability, such as Williams syndrome (WS).

**Objective:**

In adults with WS, describe predicted dietary intake, food-related problems, and associations between body mass index (BMI) and possible factors contributing to differences in weight status.

**Design:**

In this study of 82 participants (median age of 30 years, range 18–69), we cross sectionally investigated associations between BMI, predicted dietary intakes (Dietary Screener Questionnaire), food-related behaviors (Food-Related Problem Questionnaire), and anxiety (Spence Children’s Anxiety Scale). Longitudinal patterns of weight change were further studied in a subset (*n* = 41).

**Results:**

BMI variation was observed with median BMI of 27.3 kg/m^2^ (range 16.7–55.5 kg/m^2^). Several components of dietary intake deviated from recommendations in the WS cohort. When compared with WS participants with either normal or overweight BMI, WS participants with obesity had reduced daily intake of fruits and vegetables of 0.15 cup equivalents (*P* = 0.049), while participants with underweight BMI had reduced daily intake of fruits and vegetables of 0.44 cup equivalents (*P* = 0.026) and additionally had reduced intake of dietary fiber of 2.12 grams per day (*P* = 0.019). A one-point increase in the ‘preoccupation with food’ sub-score was associated with a 0.57 unit increase in BMI (*P* = 0.16), while a one-point increase in the ‘takes and stores food’ sub-score was associated with a 0.72 unit increase in BMI. In the longitudinal weight subset, a weight gain group and a weight stable group were identified. The former was associated with increased ‘takes and stores food’ sub-score but not with dietary intakes.

**Conclusion:**

We observed considerable BMI variability. While few dietary intakes were associated with BMI, increased BMI and weight gain were associated with ‘preoccupation with food’ and with ‘takes and stores food’ behavior sub-scores.

## Popular scientific summary

- Variability in BMI and patterns of weight change over time were observed in a group of adults with Williams syndrome, a rare genetic disorder associated with intellectual disability.
- Dietary intakes of individuals with Williams syndrome deviated from national recommendations; however, few of the dietary intake components were related to BMI variability.
- Selected food-related problem behaviors were found to be associated with increased BMI or higher rate of weight gain.

Healthy diet and body weight are important for long-term health and well-being. An abundance of information documents the negative health effects of having a body mass index (BMI) ≥30 kg/m^2^ (classified as obesity) or a BMI < 18.5 kg/m^2^ (classified as being underweight). Less scientific attention has been paid to the issue of weight status among adults with intellectual and developmental disability (IDD), despite studies demonstrating an increased frequency and earlier onset of obesity compared with the general population (1–3). Here we present work on one specific multisystem genetic disorder associated with IDD, Williams syndrome (WS). WS is caused by a chromosome 7q11.23 microdeletion, which produces a loss of one copy of 26–28 unique sequence genes, including the elastin gene. In terms of cognitive features, mild to moderate IDD is typical with a characteristic pattern of cognitive strengths and weaknesses. Common behavioral features include hypersociability and high rates of psychiatric comorbidities, particularly anxiety (4–7), while medical features include but are not limited to vascular stenoses, connective tissue abnormalities, distinctive facial features, dental anomalies, and multiple endocrine abnormalities (8). Infants and toddlers with WS often experience feeding difficulties, which provides a partial explanation for failure to thrive in some young children, and slow weight gain in many. In contrast to weight findings in childhood, several small series focusing on adults with WS observed a broad BMI range (8–11). The majority of adults in these series were classified as having overweight or obesity, though a small proportion with underweight BMI were observed as well. Potential dietary or behavioral factors contributing to differences in patterns of weight change over time, however, have received essentially no scrutiny to date. Examination of the WS diet is limited to a single study (11) and so far, no work exists that compares dietary intakes among persons with WS classified by BMI or sex. Likewise, there are no data that compares persons with WS to a healthy control population or which assesses the impact of food-related behavioral problems on BMI or longitudinal weight change patterns.

The objectives of this study were to investigate BMI in adults with WS and to reveal possible factors contributing to differences in weight status classifications and weight gain in adults with WS.

Our specific aims were to: 1) describe predicted intakes of selected dietary components in adults with WS and compare intakes with controls from National Health and Nutrition Examination Survey (NHANES); 2) analyze associations between BMI, predicted intake of dietary components, food-related behaviors, and anxiety in the WS study participants; and 3) for a subset of participants with WS, compare those with the highest rate of weight gain to those with stable weight over time to further explore possible factors associated with weight gain.

## Methods and analyses

### Subject recruitment

Individuals with WS and their families were recruited through a study announcement posted on websites of the Williams Syndrome Association (www.williams-syndrome.org), The Williams Syndrome Patient & Clinical Research Registry (www.williams-syndrome.org/registry), Rally with Mass General Brigham (https://rally.massgeneralbrigham.org/), or by hard copy invitation sent to personal patients of one of the authors (BRP). Eligibility criteria included the following: males or females between the ages 18 and 70, diagnosis of WS, and availability of a parent or guardian to participate in the consent process and provide medical history.

Written consent or assent, depending on legal guardianship status, was obtained from all WS participants in the study. An adult parent, family member, or legal guardian was also required to participate in the consenting procedures along with the person with WS regardless of legal guardianship status. This study was approved by the Mass General Brigham Human Research Committee.

Adults with WS participated in this study either in the Translational and Clinical Research Center at Massachusetts General Hospital (tCRC at MGH) between October 2018 and February 2020 or at a Williams Syndrome Association Family Convention (Baltimore MD July 11–14, 2018 or Minneapolis MN, November 7–9, 2019). Study recruitment was terminated at the start of the COVID-19 pandemic.

### Study procedures

A physical examination was performed by a study physician. Height and weight measurements were obtained and these were used to calculate BMI. Participants were classified into BMI categories according to the World Health Organization criteria underweight (<18.5 kg/m^2^), normal (18.5–<25 kg/m^2^), overweight (25–<30 kg/m^2^) or obese (≥30 kg/m^2^) (12). Participants were also asked to sign a medical record release form authorizing study staff to obtain historic height and weight records from their primary care provider’s office.

### Survey data

1. All participants, with a family member or guardian, completed several surveys. For the purpose of this study, we developed a health and family history questionnaire, which covered topics such as past medical history, family history, weight status over time, and medication use at the time of the study.
2. The Dietary Screener Questionnaire (DSQ), developed by the National Cancer Institute (NCI), calculates predicted mean daily intake (subsequently referred to as daily intake) using responses to 26 survey questions. Specifically, daily intakes are based on self-reported intake frequencies during the past month of multiple food and beverage item groups; frequency report options include: never, number of times last month, number of times last week, and number of times per day. Responses were analyzed using the current National Cancer Institute -recommended scoring procedures developed on data from the 2009–2010 NHANES cohort (13, 14). This scoring procedure predicts daily mean intake of selected dietary components important for the overall quality of diet, including fruits and vegetables, dairy, whole grains, fiber, calcium, added sugars, and sugar-sweetened beverages.
  DSQ food frequency responses from *N* = 2,022 adults belonging to the 2009–2010 NHANES cohort were used as a control cohort (13). The control sample size was obtained by selecting all adult cases on whom DSQ food frequency responses were available; non-pregnant and White non-Hispanic cases were analyzed to approximate characteristics of our WS cohort.
  General population daily intake recommendation and allowance values were obtained from the U.S. Department of Agriculture (USDA) and U.S. Department of Health and Human Services (HHS) Dietary Guidelines for Americans (DGA) 2020–2025, Table 4-1 (page 96), for adults consuming a 1,800–2,400 calorie per day diet (15).
3. Food and eating behaviors in the cohort were characterized using responses to the Food-Related Problem Questionnaire (FRPQ). This is a 16-item questionnaire scored on a 7-point Likert scale from 0 (never) to 6 (always) indicating the frequency of the behavior, with a Likert score ≥1 indicating at least some frequency of the behavior. Results are summarized in subscores of ‘preoccupation with food’, ‘impairment of satiety’, and ‘composite negative behavior’. The ‘composite negative behavior’ can be further divided into subscores of ‘takes and stores food’, ‘inappropriate response’, and ‘eats inedible items’ (16). The FRPQ was developed to assess eating related problem behaviors in individuals with Prader-Willi syndrome (16) but has also been used to characterize food-related problem behaviors in other genetic syndromes as Smith–Magenis syndrome and Angelman syndrome (17–19). Pica is not a feature that occurs in adults with WS, so the subscore of ‘eats inedible items’ was not included in the assessment. All other subscales were scored.
4. The Spence Children’s Anxiety Scale-Parent version (SCAS-P), a validated parent proxy-report survey (20), was used to assesses total anxiety score. The instrument allows for scoring of six subscales: panic attack and agoraphobia, separation anxiety, physical injury fears, social phobia, obsessive compulsive, and generalized anxiety disorder. The caretaker is asked to respond to each question on a four-point Likert scale for how often each anxiety item occurs (never to always).

### Weight trajectory

Data from participants who authorized access to historic heights and weights from records in their primary care provider’s office were used to generate weight (kg) over time (months) trajectory plots. Individuals with two or more weight points at least 6 months apart were included in this analysis; the terminal weight point for each subject was measured during study participation.

### Statistical analysis

Variables were assessed for normality using the Shapiro–Wilk test; log transformation was applied to skewed data. DSQ daily intake for each dietary component group was calculated as described in Methods section.

### Dietary intake in WS and in WS versus NHANES controls

Independent samples *t*-tests examined sex differences in daily intake in the WS cohort only.

Welch’s *t*-test assessed for BMI and age differences between the WS and NHANES cohorts. Linear regression analyses controlling for age assessed for daily intake differences between cohorts; this was repeated in sex-separated models also controlling for age. Bonferroni correction for the eight multiple comparisons within the whole cohort as well as the sex-specific sub cohorts, was applied to adjust the significance level (*P* < 0.00625).

### BMI associations with dietary intake including sex differences, FRPQ food behaviors and SCAS anxiety scores

Logistic regression analyses adjusted for sex and age examined BMI category associations with DSQ daily intakes as follows: obesity versus normal weight and overweight, and underweight versus normal weight and overweight.

FRPQ subscale scores were computed for each subject. Linear regression analyses adjusted for sex and age investigated associations between BMI and FRPQ subscale scores, and between BMI and frequencies of individual food behaviors.

SCAS subscale/total scores were computed for each subject. Kruskal–Wallis tests were utilized to assess for differences between BMI categories (obesity vs. normal weight and overweight, and underweight versus normal weight and overweight) and mean SCAS subscale/total scores.

### Weight trajectory modeling and analyses

A linear mixed-effects model utilizing the restricted maximum likelihood method with a diagonal covariance structure evaluated weight trajectories over time and the effects of covariates. The model included age, sex, and their interaction for fixed effects. Difference in subject starting weight was accounted for as a random effect of subject-specific intercept. An average weight change over time (kg/year) was obtained controlling for the fixed effects described here. Subject-specific slopes were obtained, and weight point specific residuals based on group mean revealed two distinct classes of weight trajectories for analysis: Group 1: ‘high gain trajectory’ was comprised individuals with a line of best fit slope of a magnitude in the top third of the cohort; and Group 2: ‘stable weight’ individuals, with a slope in the middle third of the cohort closest to zero and who did not experience two consecutive periods of gain or loss more than 2.5% of total body weight. Slopes from a third group of individuals were not used for analysis as they did not meet criteria for either Group 1 or Group 2 (see raw data for Group 1, Group 2, and Group 3 in Supplementary Fig. 1).

To explore possible factors associated with weight gain, chi-square analyses compared the two weight groups in factors such sex distribution, diagnosis of diabetes mellitus, self-reported current use of levothyroxine, or of weight-promoting medications as classified in Wharton et al. (21) and also including medroxyprogesterone. Mann–Whitney U tests were used to compare daily intake data, FRPQ (subscales and individually reported food behaviors), and SCAS scores (total and subscale) between the two weight trajectory groups. Independent samples *t*-tests investigated potential associations with birthweight.

Data were analyzed using SPSS Statistics for Windows, version 24.0 (SPSS Inc, Chicago, Ill., USA). In analyses of food behavior data STATA version 16 (StatCorp LLC, college Station, TX) was used. Figures were created using JMP Version 16 (SAS Institute Inc., Cary, NC, 1989–2021).

## Results

A total of 86 adults with WS consented to participate in this study. Thirty individuals took part in the tCRC at MGH, while 56 participated at a WSA Family Convention. Four participants were removed from all statistical analyses due to missing data for both dietary assessment and historic weight measurements; accordingly, the cohort available for detailed analyses consisted of 82 adults with WS. The diagnosis of WS was confirmed by genetic testing (fluorescent *in situ* hybridization [FISH] or chromosomal microarray analysis [CMA]) in 79 of the 82 individuals while, in the remaining three, the diagnosis was confirmed clinically by one of the co-authors (BRP), an experienced medical geneticist.

More female than male adults with WS took part in this study (*N* = 47 females; 35 males) (Table 1). There were no significant age, race, or mean BMI differences between sexes. While the male to female proportions among those in the underweight (BMI < 18.5 kg/m^2^), normal (18.5–<25 kg/m^2^), or obese (BMI ≥30 kg/m^2^) categories were comparable, there was a significantly higher percent of males with BMIs in the overweight range (BMI 25–<30 kg/m^2^: 37.1% M vs. 19.1% F) (Table 1).

**Table 1:** Characteristics of adult study participants with WS, *N* = 82

Cohort characteristics	All *N* = 82	Males *N* = 35	Females *N* = 47
Years of age, median (range)	30 (18–69)	32 (19–54)	28 (18–69)
Race white, *N* (%)	80 (97.6)	35 (100)	45 (95.7)
Race other (%)	2 (2.4)	0 (0)	2 (4.3)
Body mass index (BMI^&^), median (range)	27.3 (16.7–55.5)	27.6 (17.5–47.2)	25.8 (16.7–55.5)
BMI categories, *N* (%)			
Underweight (BMI < 18.5)	9 (11.0)	3 (8.5)	6 (12.8)
Normal weight (BMI 18.5–<25)	24 (29.3)	8 (22.9)	16 (34.0)
Overweight (BMI 25–<30)	22 (26.8)^*^	13 (37.1)	9 (19.2)
Obese (BMI ≥ 30)	27 (32.9)	11 (31.5)	16 (34.0)
Living situation, *N* (%)			
Independently	12 (14.6)	2 (5.7)	10 (21.3)
With family	47 (57.3)	23 (65.7)	24 (51.1)
Group home	12 (14.6)	5 (14.3)	7 (14.9)
Other non-independently	11 (13.5)	5 (14.3)	6 (12.7)
Zip code defined area^×^, *N* (%)			
Metropolitan/micropolitan	75 (91.5)	43 (89.6)	32 (94.1)
Small town/rural	7 (8.5)	5 (10.4)	2 (5.9)

*N* defines number of subjects.

* Male versus Female *P <* 0.05; *P-*values are generated by use of Kruskal–Wallis equality-of-populations rank sum tests for continuous variables and chi-square tests for categorical variables.

& BMI is calculated as [kilograms / (meters^2^)].

× Defined by the 2010 United States Department of Agriculture Rural-Urban Commuting Area Codes (42).

Participants with WS as a group had a mean BMI comparable to NHANES controls (kg/m^2^) (27.9 ± 8.2 WS vs. 28.7 ± 6.9 NHANES, *P =* 0.936) but were, on average, younger (32.4 years ± 11.1 WS vs. 46.1 ± 14.0 NHANES, *P <* 0.001). Mean daily intakes for selected major food groups are shown in Table 2 for the cohort with WS and for NHANES controls. Daily intakes of dairy and calcium were the only two significant intake differences revealed between the WS and NHANES cohorts. Specifically, daily intake of dairy in WS males (2.6 ± 1.1 cup equivalents WS vs. 1.8 ± 0.7 cup equivalents NHANES, *P <* 0.001), and of calcium (1292.1 ± 292.2 mgs WS vs. 1097.3 ± 232.0 mgs NHANES, *P <* 0.001) were higher than in NHANES males. A similar non-significant trend was observed in females for dairy (1.7 ± 0.6 cup equivalents WS vs. 1.5 ± 0.5 cup equivalents NHANES, *P* = 0.011) and calcium (934.0 ± 164.6 mgs WS vs. 866.6 ± 117.5 mgs NHANES, *P* = 0.017). A combined sex analysis retained significance for both intake categories, though the effects were attributed primarily to the excess in WS males (calcium *P* = 0.002; dairy *P* < 0.001).

**Table 2:** Predicted mean daily intake in subjects with WS compared with NHANES controls. Recommendations for general population daily intake obtained from Department of Agriculture (USDA) and US Department of Health and Human Services (HHS) Dietary Guidelines for Americans (DGA)^&^

Dietary component	Recommended daily intake/allowances^&^	All Mean (SD)		Males Mean (SD)		Females Mean (SD)	
		WS	NHANES	WS	NHANES	WS	NHANES
Vegetables	2.5–3	2.2 (0.6)	2.3 (0.7)	2.3 (0.6)	2.4 (0.8)	2.2 (0.6)	2.2 (0.7)
Fruits	1.5–2	0.9 (0.4)	0.9 (0.5)	0.9 (0.5)	0.9 (0.5)	0.8 (0.4)	0.9 (0.4)
Dairy	3	2.1^**^ (0.9)	1.7 (0.6)	2.6^**^ (1.1)	1.8 (0.7)	1.7^*^ (0.6)	1.5 (0.5)
Total added sugar (teaspoon equivalents/day)	≤11.1–14.7	18.9^∞^ (9.0)	16.8 (7.1)	23.2 (11.0)	18.7 (8.1)	15.7 (5.4)	15.0 (5.4)
Sweetened beverages	NA	9.0^∞^ (7.5)	8.2 (6.0)	11.7 (9.6)	9.7 (6.5)	7.0 (4.8)	6.8 (5.2)
Whole grains	3–4	0.7 (0.4)	0.7 (0.4)	0.8 (0.4)	0.8 (0.4)	0.7 (0.3)	0.7 (0.4)
Dietary fiber	25–34	15.5^∞^ (3.0)	16.0 (3.4)	16.7 (3.0)	17.1 (3.4)	14.6 (2.7)	14.5 (3.0)
Calcium	1,000	1089.5^**,∞^ (289.0)	982.1 (226.6)	1292.1^**^ (292.2)	1097.3 (232.0)	934.0^*^ (164.6)	866.6 (117.5)

All *P*-values adjusted for age by linear regression.

WS versus NHANES differences that reached statistical significance are indicated by an asterisk.

* *P* < 0.050 (uncorrected *P* for multiple comparisons).

** *P* < 0.00625 (Bonferroni corrected *P* for eight multiple comparisons).

∞ *P* < 0.00625 WS within cohort male versus female difference.

& Based on Dietary Guidelines for Americans 2020–2025 recommendations for 1,800–2,400 calorie diet (15).

Sex analyses within the WS cohort (Table 2) demonstrated that males with WS, in comparison to females with WS, had higher mean daily intakes for the following components: added sugars (*P* < 0.001), sugar-sweetened beverage serving equivalents per day (*P* = 0.050), dairy (*P <* 0.001), dietary fiber (*P* = 0.001), and calcium (*P <* 0.001).

Comparison to national dietary recommendations from the 2020 Dietary Guidelines for Americans (DGA) (15) showed that mean daily intakes of added sugars for both the WS and NHANES groups were greater than allowance range (Table 2). Notably, 51% of individuals with WS, and 46% of NHANES adults, consumed more than the daily allowance of 11.1–14.7 tsp equivalents of total added sugar per day. Mean daily intakes of fruits, whole grains, and fibers for adults with WS were lower than DGA recommendations, as were the NHANES mean intakes. 87.8% of NHANES and 82.6% of WS adults consumed less than the recommended daily intake of fruits, while 96% of NHANES and 91.9% of WS adults did so for vegetables.

WS participants with obesity (BMI ≥ 30), when compared with WS participants who had either normal weight or overweight (BMI 18.5–<30), had a lower mean intake of fruits and vegetables of 0.15 cup equivalents per day, (95% confidence interval [CI] [0.01, 0.11], *P* = 0.049). The group of WS participants with an underweight BMI (BMI < 18.5) when compared with the groups with normal and overweight, had a reduced mean intake of fruits and vegetables of 0.44 cup equivalents per day, (95% CI [0.05, 0.82], *P* = 0.026); they also had a reduced intake of dietary fiber of 2.12 g/day (95% CI [0.36, 3.88], *P* = 0.019). No significant associations between BMI category and mean intake of dairy, added sugars or whole grains were detected.

FRPQ subscale score analyses showed the total ‘preoccupation with food’ sub-score was 5.87 (standard deviation [SD] 3.75) of a maximum score of 18, while the total ‘impairment of satiety’ sub-score was 10.33 (SD 4.15) of a maximum score of 30. Examination of specific items that comprise the ‘impairment of satiety’ subscale reveals a high frequency of selected food behaviors. The majority of adults with WS (70%) were reported as displaying at least some frequency of the behavior, ‘if given the opportunity, eats more than a standard sized meal’. For the ‘takes and stores food’ subcategory, the total sub-score was 3.83 (SD 3.93) of a maximum score of 18; 87% of adults were reported displaying some frequency of helping themselves to food that they should not have, a specific item that comprises this subscale.

In a linear regression model adjusted for sex, a one-point score increase in the FRPQ ‘preoccupation with food’ subscore was associated with a 0.57 unit increase in BMI, (95% CI [0.11, 1.02], *P* = 0.016), while a one-point increase in the ‘takes and stores food’ subscore was associated with a 0.72 unit increase in BMI, (95% CI [0.30, 1.15], *P* = 0.001). No significant association was found between BMI and the ‘impairment of satiety’ sub-score.

Across the subset of participants with available historic weight data (*N* = 41), weight increased at an average rate of 0.08 kg/month (95% CI [0.05, 0.12], *P <* 0.001). For these 41 participants, mean duration of weight trajectory data was 64.43 months (range 6–228); mean age at starting weight point was 28.8 ± 9.8 years; and mean age at terminal point was 34.4 ± 10.8 years. Compared with males, the rate of weight increase was greater in females by 0.10 kg/month (95% CI [0.06, 0.13], *P* < 0.001).

**Fig. 1:**
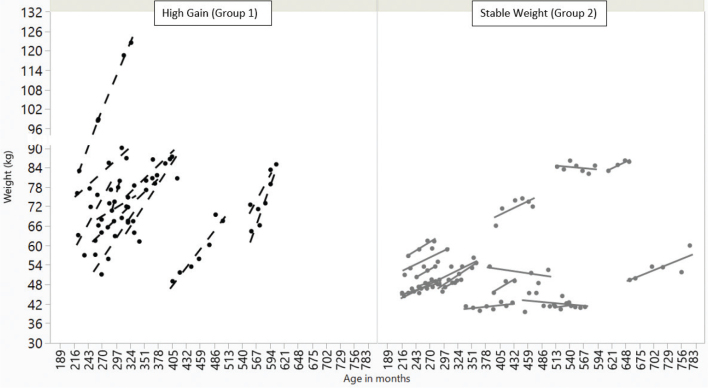
Weight trajectories (• = actual weight) in adults with WS. Group 1, panel on left, (*N* = 13) shows adults with high weight gain during time period of available data, while Group 2, panel on right, (*N* = 17) shows those with more stable weight over time. Group 1 scored higher on the FRPQ subscale “takes and stores food” but no other differences such as M:F ratio, average age, or weight promoting medications were observed. Lines = line of best fit.

Historic weight subset participants were classified into distinct weight trajectory groups (Fig. 1) based on mixed model analysis as follows: Group 1 (high weight gain trajectory, *N* =13); Group 2 (stable weight, *N* = 17); and Group 3 (unable to classify into Group 1 or 2, *N* = 11). Individual subject’s slopes in Group 1 ranged from 0.11 to 0.60 (kg/month), while in Group 2 ranged from 0.02–0.09 (kg/month).

Groups 1 and 2 had a comparable percent of males (Group 1: 38.5% male; Group 2: 33.3% male; *P* = 0.778). Groups 1 and 2 were also not significantly different in average age at starting and terminal measurement (starting age: Group 1: 30.7 ± 11.7 vs. Group 2: 26.8 ± 10.1, *P* = 0.397; terminal age: Group 1: 36.6 ± 12.4 vs. Group 2: 32.9 ± 9.6, *P* = 0.267). Group 1 scored higher on the FRPQ subscale ‘takes and stores food’ with a median score of 3.00 for Group 1 and 0.00 for Group 2, *P* = 0.003. Additional analyses revealed no significant differences between Groups 1 and 2 including: frequency of diabetes mellitus and use of levothyroxine; frequencies of reported weight-promoting medications (*P* = 0.339); percentages of individuals reporting first or second-degree family members with overweight/obesity (*P* = 0.547); or birth weight (g) (*P* = 0.487). Likewise, no differences were noticed between Group 1 and Group 2 in dietary intakes, or SCAS scores (see Supplementary Table 1).

To view each subject’s actual weight points included for analysis in spaghetti plot format, see Supplementary Fig. 1.

## Discussion

In a cohort of adults with WS, we examined BMI cross sectionally and, in a subset, longitudinally. We observed considerable BMI variability and sought to identify contributory factors. These factors fell into two broad categories: dietary differences based on a food frequency questionnaire (DSQ) and food-related behaviors (based on FRPQ). Results from the DSQ revealed deviations from dietary recommendations for several components (15) in the WS cohort that are indicative of poor diet quality. Differences in dietary intake between BMI categories were revealed only for fruit and vegetable intake for individuals with either obesity or underweight BMI, and additionally in dietary fiber intake for individuals with underweight BMI; no other distinctions were found. Analysis of endorsement of food-related behavioral issues based on the FRPQ was more provocative and raised the possibility that problems in the behavioral domain, not previously appreciated in adults with WS, may be associated with BMI.

### Dietary intakes

On average, the WS cohort, as well as the NHANES controls, consumed less than the recommended intake of fruits, vegetables, whole grains, and dietary fiber, but greater than the allowance guidelines for added sugars (13, 15). This pattern has also been observed in other adult populations with IDD (22–24).

Low intake frequency of fruits and vegetables in WS, and lower plasma carotenoids compared with other IDD groups, has previously been described in a Norwegian cohort of adolescents and adults with WS (11). Plasma carotenoids levels, a biomarker of fruits and vegetable intake, tended to be lowest in persons with WS whose BMI was <18.5 (underweight). This association of low carotenoids with low BMI is in accordance with the present data. However, all our WS subjects, regardless of BMI classification, had a lower daily intake of fruits and vegetables than recommended by the DGA (15).

The mean daily intake of dietary fiber in our WS cohort was reduced by approximately 50% of the DGA recommendation (15). A common problem, estimated to complicate the lives of half of individuals with WS, is chronic or recurring constipation that is likely related to inadequate dietary fiber intake (25). Though the food frequency intake data does not capture information on fiber supplements, not all supplements prevent constipation and furthermore, non-psyllium containing products appear not to provide the same broad range of health benefits as dietary fiber (26).

Among general population adults, numerous studies indicate that a poor-quality diet, one that is high in refined carbohydrates and low in fruits and vegetables, increases the risk of various medical problems including but not limited to obesity, diabetes mellitus, high blood pressure, and stroke (27–29). It is possible that these problems, already well-known to complicate WS, may be amplified by poor diet quality. While dietary management guidelines exist for Prader–Willi syndrome and Down syndrome, which include recommendations for reduced intake of simple carbohydrates, increased dietary fiber and fruits and vegetables (24, 30–32), no published guidelines currently exist for individuals with WS.

One final result to expand on is that the daily calcium intake in the WS males exceeded the DGA recommendation and was also significantly higher than intakes for NHANES males and WS females. Since persons with WS are at an increased risk of hypercalcemia, especially during infancy, dietary calcium intake is often moderated by caregivers/families (most commonly as limiting dairy products) (33) and this can turn into a lifelong pattern (clinical observation, BRP). Yet our results suggest this is uncommon, especially in WS males, some of whom are consuming more calcium from food sources than is recommended. If this finding is confirmed in an independent cohort, it suggests that dietary calcium intake should be routinely assessed in adults with WS before supplemental calcium is recommended.

### BMI (cross-sectional data)

BMI among the WS adults in this study ranged from a low of 16.7 kg/m^2^ to a high of 55.5 kg/m^2^ (median 27.3). The majority of the cohort (60%) was classified as having either overweight or obesity, but it is important to also point out that 11% of our study participants had a BMI in the underweight range (BMI ≤18.5).

Four other WS series on comparably aged adults demonstrated a similar frequency of subjects with a BMI ≥ 25, ranging from 49 to 65% (8–11). In terms of underweight individuals, Nordstrom et al. reported 13% of the WS cohort met criteria for being underweight, which is comparable to the 11% we observed. The other three studies had 0–7% of participants with a BMI < 18.5 but were focusing primarily on the association of diabetes mellitus and WS.

BMI comparisons to other disorders with IDD is challenging due to methodologic differences and modest sample sizes. Excluding comparison to adults with Down syndrome, a group well-known to have a high prevalence of obesity, limited data suggests similar BMI distributions among adults with WS, adults with IDD of unknown etiology, and adults with autism spectrum disorder. However, being underweight may more commonly complicate the diagnosis of WS especially when compared with those with IDD of unknown etiology (34, 35). Chronically underweight individuals are at an increased risk of a variety of medical concerns including decreased bone density, impaired immunity, selected nutrient deficiencies, and fatigue among others (36). Accordingly, we recommend that further work in adults with WS be performed on the issue of underweight status in the hopes of identifying potentially remediable medical and/or psychological factors.

### Food behavior

We are the first report on food-related behaviors, using the FRPQ, in adults with WS. Prevalent problematic food behaviors in WS (Table 3) included talks about food (91%), helps themselves to food they should not have (87%), responds negatively if denied food (74%), and eats more than a standard sized meal (70%). Furthermore, a stronger endorsement of food preoccupations, and of taking and storing food, are associated with an increase in BMI.

**Table 3:** Responses to individual questions in the Food-Related Problems Questionnaire (FRPQ); *N* = 78 subjects with WS

Individual FRPQ questions	Percent of sample with report of food behavior^1^ (%)	Average Likert scale response^1^ 1–6 (SD)
**Preoccupation with food**		
Compares size of meal with other	59	1.80 (1.34)
Talks about food	91	3.23 (1.61)
Associates people or places and/or occasions with specific food	73	3.20 (1.42)
**Impairment of satiety**		
Still feels hungry after a normal sized meal	71	2.05 (1.30)
Goes without food if feeling tired, ill or upset	74	2.07 (1.34)
Shares food with others	87	2.46 (1.32)
Describes feeling full	97	4.20 (1.09)
Given the opportunity, eats more than a standard sized meal	70	2.48 (1.37)
**Takes and stores food**		
Helps themselves to food they should not have	87	2.75 (1.45)
Hides or hoards food	39	2.83 (1.46)
It is necessary to lock food away	23	3.11 (1.65)
**Inappropriate response**		
Accepts explanation if meal is delayed	100	5.12 (1.08)
Responds negatively if denied food	74	3.01 (1.68)
Become upset or angry if a meal includes a food not expected	60	2.35 (1.41)

1 Including individuals responding ≥ 1 on the 7-point Likert Scale, frequency of any occurrence of behavior.

Table adapted from Alaimo et al. (17).

We compared FRPQ findings from the WS cohort with other adults with IDD reported in the literature, namely: 1) adults with Prader–Willi syndrome (PWS); 2) adults with Smith–Magenis syndrome (SMS) using adult data kindly extracted by Dr. Sarah Elsea from Alaimo et al. (17); and 3) adults in a generalized intellectual disability group. For the ‘food preoccupation’ subscale, our WS cohort scored higher on average than a generalized IDD group but lower than PWS and SMS adults (IDD = 3.8 ± 2.4, WS = 5.9 ± 3.8, PWS = 8.1 ± 2.0, SMS = 8.6 ± 3.4) (16, 17). We also observed several more positive highly prevalent food behaviors in WS; for instance, all participants could accept an explanation if a meal is delayed, and 87% can share food with others. These behaviors point toward the social aspects of meals and this a strength in persons with WS.

The food-related behavior findings in WS suggest that the development of tailored weight reduction interventions for adults with WS might need to incorporate strategies on how to tackle and avoid some of these problematic behaviors. This perspective is supported by a small study in adolescents and young adults with Down syndrome. The largest weight loss occurred in the group where parents received education in behavioral strategies in addition to individual diet and exercise plans, while parents in the comparison group only received individual diet and exercise plans (37). Behavioral interventions for food-related behaviors have also been described in Prader–Willi syndrome with success, including considerations for managing hyperphagia with cognitive behavioral therapy (38, 39). The benefit of layering behavioral interventions particularly to address preoccupation with food needs to be investigated in larger studies on persons with WS and a role for pharmacotherapy to assist in the management of food preoccupations merits consideration as well.

### Weight trajectory

A dearth of information on weight trajectories in adults with IDD exist and, to our knowledge, this study is the first to investigate individual trajectories. Those in Group 1 (Fig. 1) experienced a mean rate of gain of 3.5 kg per year, and nearly all these individuals were classified as having obesity at time of study measurement (final weight point). In a search for potential causative differences between those with the highest weight gain (Group 1) and those in the stable weight group (Group 2), we did not identify any significant differences (including in male to female ratio, frequency of diabetes mellitus, use of weight promoting medications, etc.). However, an increased score for the FRPQ subscale ‘takes and stores food’ was noticed in Group 1 compared with Group 2 (Supplementary Table 1).

To our knowledge, only a single previous study examined weight change over time in adults with IDD (34). Ptomey and colleagues reported mean annualized percent weight change of −0.2, 1.6, and 2.2% for several hundred adults with Down syndrome, autism spectrum disorder, and IDD, respectively (34). When examining percent weight change in the present WS cohort, we find a mean 2% annualized weight change. However, due to methodologic differences in data collection, it is difficult to compare the weight change findings of Ptomey et al. (34) with ours.

In our view, further investigation of adult weight trajectories is a rich area for future study as a means to identify biological, behavioral, or environmental factors that contribute to weight gain, that might prevent weight gain, or that cause and perpetuate being underweight. And though it was not a focus of our current analyses, patterns of high weight fluctuations occurred in several of our subjects [see unclassified group (Group 3) in Supplementary Fig. 1]. Scrutinizing the medical and psychological well-being of subjects at the time of these fluctuations may reveal predictable events, which could allow preventatives to be put in place to mitigate fluctuations.

### Limitations and strengths

We elected to administer the DSQ based on several strengths of the instrument, including excellent validation in a large general population cohort by comparison to 24-h recall data (14) and simple, easy to understand question content and format making it suitable for persons with WS. In addition, the DSQ allowed for comparison to a large control group (13). However, there are distinct limitations to the DSQ compared with a comprehensive assessment of dietary intake, first among which is underreporting of unhealthy foods and overreporting of healthy foods, with a potentially stronger effect in persons who have overweight and obesity (40). While these limitations are inherent to any measure that collects self-reported dietary intake information, particular concerns can be raised about information from adults with WS. Reassuringly, another study of adults with WS (11) demonstrated good agreement between frequency of reported intake and measured biomarkers with dietary implications. Similarly, in this study, DSQ information was captured by parent/caregiver proxy-report combined with input from the individual with WS. Since the majority (57%) of our subjects resided at home with parent/caregivers, we believe this enhanced accuracy but appreciate this could be a limitation for individuals living independently or in a group living situation.

In terms of the FRPQ, it has been previously used in adults with PWS and SMS in multiple living situations (16, 17, 41). The recommended approach to FRPQ interpretation is to tally and compare subscale scores (16). We chose to pay particular attention to individual question responses so that we could obtain greater granularity into food-related behaviors among adults with WS. Findings from this exploratory report on food behaviors may guide future research into behavioral contributions to weight variability.

In terms of sample bias, it is possible that adults with WS and their families who have experienced issues with either low or excess body weight were more likely to take part in this study, which was described to families as focusing on factors contributing differences in body shapes and sizes. This bias does not invalidate the study’s findings but does limit their generalizability to the universe of adults with WS who may not have concerns about body shape.

Lastly, analyses of weight trajectories were performed on relatively small samples sizes and were based on retrospectively collected medical records; therefore, data differed between subjects on variables such as ages for beginning and terminal weight points; duration of weight trajectory plots; time between measurements; and equipment used to obtain measurements. While a mixed model accounts for these factors, larger sample sizes and longer time intervals of data collection are needed to investigate weight trends and identify contributing dietary and non-dietary factors, including food behavior problems.

## Conclusions

Considerable variation in BMI was observed among adults with WS. Mean daily intakes of fruits, vegetables, and dietary fiber in WS adults, as well as in NHANES controls, were below national dietary guideline recommended ranges. Within our WS cohort, those with a BMI in either obesity or underweight category had decreased intake of fruits and vegetables compared with normal/overweight individuals while, additionally, individuals in the underweight category had decreased dietary fiber intake. These deviations from recommended intakes are an indicator that diet quality should be further evaluated in adults with WS.

When assessing food behaviors, increased BMI and rate of weight gain were associated with increased preoccupation with food subscores and with a higher frequency of taking and storing food. For adults with WS and obesity or rapid weight gain, attention to problematic food behaviors may be an important aspect to consider and address.
