# Supplementary material for: Body mass index variation in adults with Williams syndrome: associations with predicted dietary intake and food behaviors
Source: Food Nutr Res. 2023 Jun 23;67:10.29219/fnr.v67.9321. doi: 10.29219/fnr.v67.9321 (PMC10335094; doi:10.29219/fnr.v67.9321)

Supplemental Table 1. Comparison of Weight trajectory Group 1 (High Weight gain) versus Group 2 (Stable Weight) responses on Dietary Screener Questionnaire (DSQ), Food-Related Problem Questionnaire (FRPQ), and Spence Children's Anxiety Scale (SCAS).

DSQ Variables	Mean (SD) Group 1	Mean (SD) Group 2
Vegetables (cup equivalents/day)^	1.3 (.4)	1.4 (0.3)
Fruits (cup equivalents/day)^	0.9 (0.4)	0.9 (0.3)
Dairy (cup equivalents/day) ^	2.2 (0.9)	1.9 (0.9)
Total added sugar (teaspoon equivalents/day)^	16.8 (8.2)	18.2 (8.7)
Sweetened beverages (teaspoon equivalents/day)^	8.2 (8.3)	9.2 (9.2)
Whole grains (ounce equivalents/day)^	0.9 (0.4)	0.7 (0.4)
Dietary Fiber (grams/day)^	16.2(3.1)	15.8 (3.1)
Calcium (milligrams/day)^	1078.0 (251.4)	1022.5 (310.2)
**FRPQ Subscales**	**Median (range) Group 1**	**Median (range) Group 2**
Food Preoccupation~	2 (1-4)	1 (0-6)
Impairment of Satiety ~	4 (2-7)	3 (1-4)
Takes and stores food ~ **	3 (1-5)	0 (0-6)
**SCAS Total and Subscales**	**Median (range) Group 1**	**Median (range) Group 2**
OCD ^	1.00 (0.00-4.00)	1.00 (0.00-13.00)
Panic/ Agoraphobia^	3.00 (1.00-7.00)	1.50 (0.00-10.00)
Separation^	3.00 (0.00-8.00)	2.00 (0.00-10.00)
Physical Injury ^	3.00 (0.00-11.00)	3.50 (0.00-13.00)
Social Phobia ^	3.00 (0.00-11.00)	3.00 (0.00-11.00)
Generalized^	6.00 (2.00-11.00)	5.50 (0.00-9.00)
Total Anxiety Score ^	19.00 (4.00-38.00)	17.50 (0.00-47.00)

^*p-values based on independent samples t-test*

~*p-values based on Mann-Whitney U nonparametric test*

* *Male vs Female *p<.005; p*-values are generated by use of Kruskal-Wallis equality-of-populations rank sum tests for continuous variables and chi square tests for categorical variables.

DSQ = Dietary Screener Questionnaire

FRPQ = Food-Related Problem Questionnaire

SCAS = Spence Children's Anxiety Scale

Supplemental Figure 1. Individual weight points collected at least 6 months apart on subjects with WS (N = 41). Group 1 (N = 13), panel on left, shows adults with high weight gain during period of available data; Group 2 (N=17), panel on right, shows those with more stable weight over time; and Group 3 (N = 11) are those unable to be classified into Group 1 or 2.

Supplemental Figure 1.

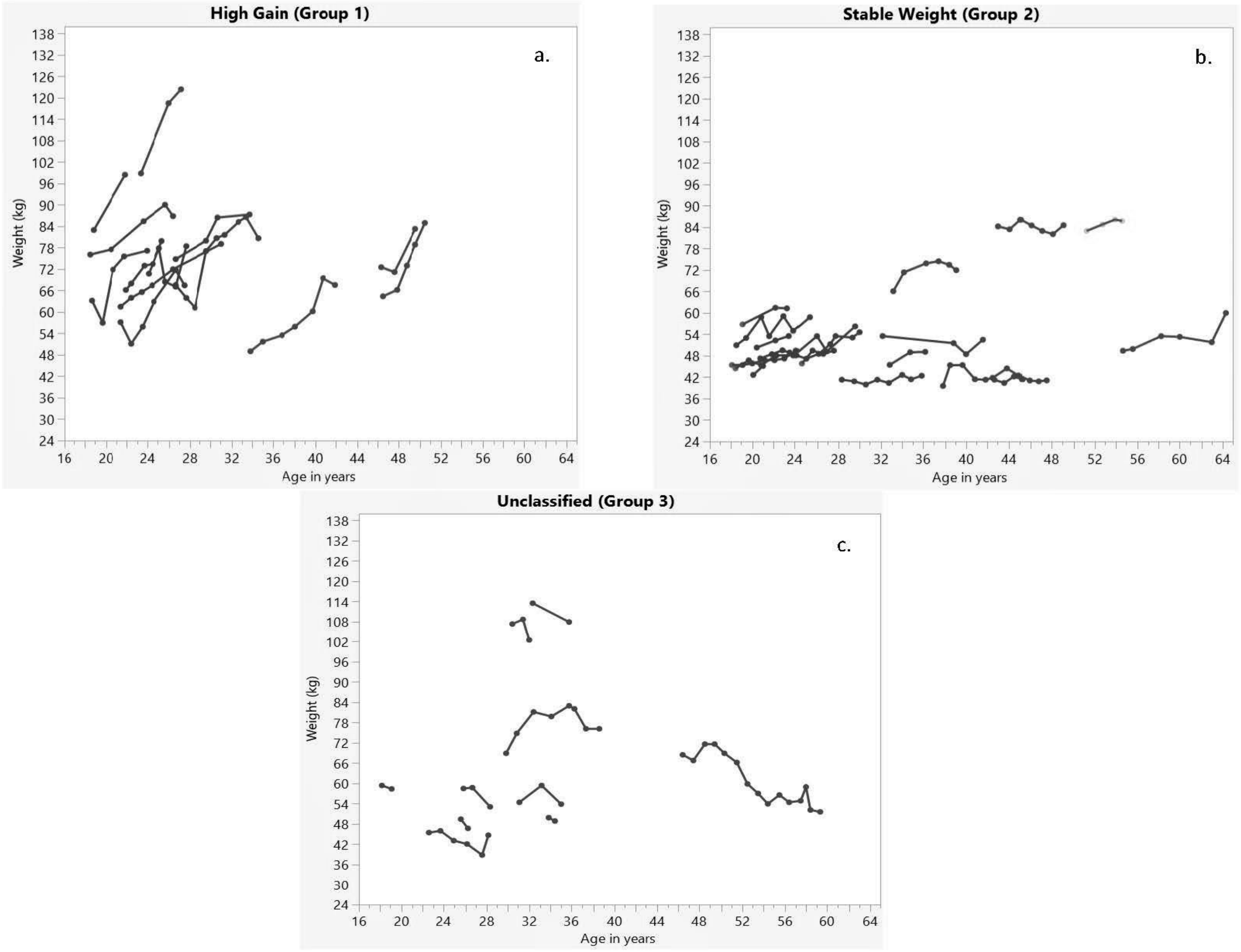

Supplement: Supplementary file 1 [file FNR-67-9321-s001.pdf]
